# *Schistosoma mansoni* experimental infection in *Mus spretus* (SPRET/EiJ strain) mice

**DOI:** 10.1051/parasite/2013027

**Published:** 2013-08-29

**Authors:** Luis Pérez del Villar, Belén Vicente, Purificación Galindo-Villardón, Andrés Castellanos, Jesús Pérez-Losada, Antonio Muro

**Affiliations:** 1 Laboratorio de Inmunología y Parasitología Molecular, CIETUS, Facultad de Farmacia, Universidad de Salamanca 37008 Salamanca Spain; 2 Instituto de Investigaciones Biomédicas de Salamanca (IBSAL) Salamanca Spain; 3 Departamento de Estadística, Universidad de Salamanca 37008 Salamanca Spain; 4 Centro de Investigación del Cáncer, Universidad de Salamanca-CSIC 37008 Salamanca Spain

**Keywords:** *Schistosoma mansoni* infection, *Mus spretus*, Immunological phenotypes, Hematological phenotypes

## Abstract

Most *Schistosoma mansoni* experimental infections are developed in several inbred strains of *Mus musculus* as definitive host. In contrast, *Mus spretus* is unexplored in *Schistosoma* infection studies. *Mus spretus* provides a high variation of immunological phenotypes being an invaluable tool for genetic studies and gene mapping. The aim of this study is to characterize hematological and immunological responses against *Schistosoma mansoni* infection in *Mus spretus* (SPRET/EiJ strain) *vs. Mus musculus* (CD1 strain) mice. Nine weeks after cercarial exposure, animals were perfused and the parasite burden was assessed. The parasitological data suggests that SPRET/EiJ mice tolerate higher parasite loads compared to CD1 strain. In addition, hematological parameters measured in *Mus spretus* group showed a significant increase in granulocytes population in early stages of infection compared to the CD1 cohort. Meanwhile, CD1 presented higher levels of lymphocytes and IgG1 in the late stages of *S. mansoni* experimental infection.

## Abstract (fr)

La plupart des infections expérimentales à *Schistosoma mansoni* sont développées chez *Mus musculus* comme hôte définitif. Au contraire, *Mus spretus* est une espèce de souris inexplorée dans les infections expérimentales á *Schistosoma*. *Mus spretus* offre une grande variation de phénotypes immunologiques, ce qui est un outil essentiel pour les études génétiques et la cartographie des gènes. L’objectif de cette étude est la caractérisation de la réponse hématologique et immunologique contre l’infection à *Schistosoma mansoni* chez *Mus spretus* (souche SPRET/EiJ) comparée à *Mus musculus* (souche CD1). Neuf semaines après l’exposition aux cercaires, les animaux ont été perfusés et les paramètres parasitologiques ont été obtenus. Les données parasitologiques suggèrent que la souche SPRET/EiJ tolère des charges parasitaires plus élevées que la souche CD1. Les paramètres hématologiques mesurés chez SPRET/EiJ ont montré aussi une augmentation significative de la population des granulocytes dans les premiers stades de l’infection par comparaison à la cohorte CD1. Cependant, la souche CD1 a présenté des niveaux plus élevés de lymphocytes et IgG1 dans les stades tardifs de l’infection expérimentale à *S. mansoni*.

## Introduction

Schistosomiasis remains one of the most important parasitic diseases affecting over 200 million human beings and causing 200,000 deaths per year [24]. However, the pathology caused by *Schistosoma* spp. infection varies widely depending on the intensity of infection and ecological factors. These issues contribute toward the differential global infection and mortality rates [2]. Furthermore, schistosomiasis susceptibility is influenced by multiple genes as well as by gene-gene and gene-environment interactions [6]. In experimental infections, inbreed mouse strains develop different degree of *Schistosoma* pathology; among these mouse strains, CBA/2J and C3H strains develop significantly higher hepatic pathology than C57BL/6J [5, 23]. At the late stages of experimental infections, the decrease of peripheral neutrophils is associated with an increase of lesion size and fibrosis in CBA mice, whereas those effects are minimal in C57BL/6 strain, indicating that neutrophils play a regulating role for granuloma formation [8]. Furthermore, enhanced neutrophil apoptosis has been reported in hepatosplenic schistosomiasis in humans [1]. Thus, identifying relevant hematological phenotypes involved in schistosomiasis susceptibility provides a useful insight into its pathogenesis in humans [3, 21].

*Mus spretus* have been the subject of various inquiries covering biometrical and morphological analyses [28]. Phylogenetic studies of mitochondrial D-loop sequences let to distinguish *M. spretus* from *M. spicilegus* and *M. musculus* as different species [11]. In fact, strains derived from wild *M. spretus* mice (i.e. SPRET/EiJ) show different divergent phenotypes and higher genetic variability than other common laboratory strains derived from *M. musculus*. Due to this phylogenetic difference, *M. spretus* mice have been useful for dissecting the genetic architecture of different complex traits including obesity, cancer, and infectious diseases [12, 22, 25, 26]. However, information about the susceptibility of *M. spretus* to experimental *S. mansoni* infection is not available. To determine whether there were specific variations in immunological and hematological responses against *S. mansoni* infection, we infected *M. spretus* mice (SPRET/EiJ*),* and compared their immunological response and infection parameters with those of *Mus musculus* (CD1) mice.

## Materials and methods

### Parasite and mice

*Mus spretus* (SPRET/EiJ) and *Mus musculus* (CD1) mice five-to six-week-old were purchased from the Jackson Laboratory and maintained in the Animal Facility at the University of Salamanca. All animals were treated according to the provisions of the current European law on animal experimentation. Cercariae of *S. mansoni* were obtained from infected *Biomphalaria glabrata* snails breeding in the Laboratory of Immunological and Molecular Parasitology, CIETUS, at the University of Salamanca. Forty mice (10♂ and 10♀ SPRET/EiJ and similarly 10♂ and 10♀ CD1) were included in the experiment. Each mouse was infected subcutaneously with 150 *S. mansoni* cercariae. Blood samples were collected at 0, 3, 6, and 9 weeks after infection. Animals were perfused at 9 weeks after infection and adult male and female parasites were counted with a dissecting microscope (10×) as previously described [20]. At the time of perfusion, small intestines and livers were collected and digested in 4% KOH for measuring the number of eggs deposited in these organs. Macroscopic lesions in the liver were quantified as granuloma affected surface per cm^2^ in each animal using the Image J software [18].

### Hematological analysis and quantitation of serum immunoglobulins

Fifty microliters of mouse blood was collected in EDTA-coated tubes (Vacutainer®), then mixed and analyzed using the HEMAVET system®. The measurement of specific antibodies (IgG, IgG1, and IgG2a) against the Specific Worm Antigen Product (SWAP) was performed using an indirect ELISA. The Specific Worm Antigen Product (SWAP) was obtained as previously described [13]. The results were expressed as means of the optical density from all the animals of each group *plus* the standard error (SEM).

### Data analysis

Statistical significance of parasitological data was analyzed by Kruskal-Wallis test. Differences with a *p*-value <0.05 were considered as statistically significant. All data were analyzed with the statistical software SPSS for Windows 11.5 (Lead Technologies), and visually displayed using the Lattice Package developed in R [17].

## Results and discussion

Several studies have reported that *Mus spretus* are highly resistant mice to inflammation process [9, 14, 27]. Thus, *Mus spretus* have been proposed as useful model to identify genetic regions contributing to differences in immune response and inflammation. Therefore, the aim of the present study was to explore the hematological features and the immunophenotypes associated to resistance/susceptibility to schistosomiasis in SPRET/EiJ mice. The first finding related to the resistance/susceptibility to the experimental *S. mansoni* infection was the difference on infection rates. Our results indicated that SPRET/EiJ mice tolerate *S. mansoni* infection better than CD1 strain*.* Total worms recoveries were higher in SPRET/EiJ than in CD1 mice (Table 1); and trapped eggs in intestine and hepatic tissues were significantly higher in SPRET/EiJ too. We also found a higher percentage of macroscopic lesions caused by *S. mansoni* eggs in SPRET/EiJ mice compared with CD1 mice (Table 1)*.* The evaluation of macroscopic granulomatous lesions was positively correlated with the intensity of infection useful for evaluating *S. mansoni* experimental infections [19]. Therefore, there was no apparent association between worm and tissue eggs burdens presented by SPRET/EiJ and its resistance to inflammation process. It should be noticed that the high intensity infection rate was also observed in C57BL/6 that is considered as resistant mouse strain to develop the severe form of schistosomiasis [16]. Concerning the effect of the mouse gender on parasitological data, we did not observe any statistical differences in any strain of mice.

**Table 1.** Worm recovery, eggs counts in hepatic and intestinal tissues, and granulomatous lesions of *Mus spretus* (SPRET/EiJ strain) and *Mus musculus* (CD1 strain) mice.

		Worm recovery			Egg density		
Sex	Mice	Male	Female	Total	Liver	Intestine	Lesions/cm^2^
Female	SPRET/EiJ	21.0 (9.8)*	31.8 (18.1)*	52.8 (27.8)*	11571.3 (5731.5)*	15154.8 (8830.4)*	44.5 (1.4)
	CD1	4.8 (1.7)	5.2 (2.0)	10.0 (3.7)	4185.2 (1585.3)	2600.6 (1367.5)	38.6 (4.7)
Male	SPRET/EiJ	18.0 (6.1)	21.0 (7.5)	38.3 (13.4)	17749.3 (5048.9)	33206.3 (14678.7)	49.6 (2.0)*
	CD1	10.2 (2.3)	11.4 (2.4)	21.6 (4.7)	6693.6 (940.5)	6440.2 (2314.5)	35.8 (2.1)

All results presented as: Mean (SEM). *p < 0.05.

**Figure 1.**
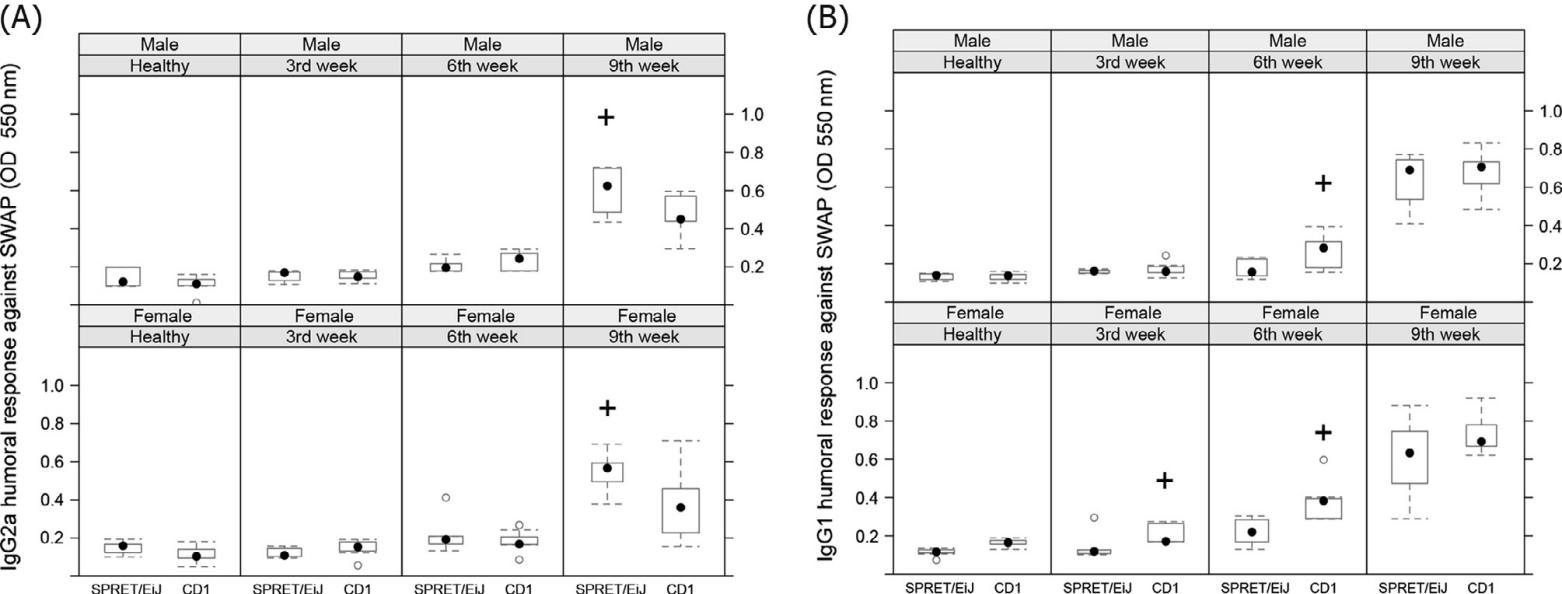
Boxplot graph displaying the levels of IgG2a (A) and IgG1 (B) along *Schistosoma mansoni* infection, according to sex and strain of mice. ^+^*p* < 0.05.

In this study, we also analyzed the immunological and hematological profiles presented during *S. mansoni* infection in SPRET/EiJ and CD1 mice*.* The variables studied were white blood cell counts, and IgG1 and IgG2a isotype-specific subclasses. Regarding the humoral response, our results show a typical progressive enhancement on antibodies levels against the SWAP during *S. mansoni* experimental infection in both subtypes of immunoglobulin analyzed. Specifically, SPRET/EiJ mice showed an increase of IgG2a levels compared to CD1 at 9 weeks post-infection in both sexes (*p* < 0.05) (Figure 1A). On the other hand, female CD1 mice showed significant higher IgG1 levels at 3 and 6 weeks after infection compared with female SPRET/EiJ mice (*p* < 0.05). Furthermore, significant higher IgG1 levels were also found in CD1 male mice compared to their SPRET/EiJ counterparts at 6 weeks after infection (*p* < 0.05) (Figure 1B). These results suggested that CD1 mice would present a stronger Th2 immune response against *S. mansoni* compared to SPRET/EiJ mice.

We hypothesized that the permissiveness of SPRET/EiJ mice to *S. mansoni* infection could be mediated by cell immune response in initial phase of infection. Therefore, we have also monitored blood cells parameters after 0, 3, 6, and 9 weeks during the *S. mansoni* infection in both mouse species. We noticed that SPRET/EiJ mice differ from CD1 in the hematological response. Interestingly, SPRET/EiJ presented high levels of peripheral blood granulocytes at 3 weeks after infection (Table 2). Blood granulocytes play a critical role in host defense mechanisms against invading pathogens; they are rapidly recruited to the infection site. In addition, these cell types are present in the initiation and maintenance of chronic allergic inflammation, and they could play a protective role in the immune response against *S. mansoni* [15]. In fact, published studies have shown that mast cells and eosinophils are able to produce IL-4 and modulate granuloma formation in *S. mansoni* infection [7, 10]. It has also been confirmed that SPRET/EiJ mice show a high level of resistance to infection of other pathogens like *Salmonella enterica* where macrophages and neutrophils also play a critical role [4]. Previous studies showed that SPRET/EiJ strain is also strongly resistant to inflammation induced either by cytokines or by bacterial products [27]. Regarding the hematological levels at 6 weeks after infection, we could observe that higher levels of neutrophils, eosinophils, and basophils continue to be associated to SPRET/EiJ strain, meanwhile, higher levels of lymphocytes populations were significantly associated to CD1 mice (*p* < 0.05) (Table 3). We also noted that total WBCs and lymphocytes populations were significantly decreased in SPRET/EiJ strain at 9 weeks after infection (*p* < 0.05). Therefore, despite attenuation of lymphocytes populations in SPRET/EiJ mice at the late stage of infection, SPRET/EiJ mice showed an enhanced tolerability to *S. mansoni* infection probably due to improved functions of the innate immune cells such as neutrophils in early time of *S. mansoni* infection. Finally, with respect the effect of mouse gender on hematological data, we did not observe any statistical significant differences in any species of mice.

**Table 2.** Summary of granulocytes count in *Mus spretus* (SPRET/EiJ strain) and *Mus musculus* (CD1 strain) mice during *Schistosoma mansoni* infection.

Time point	Sex	Mice	Neutrophils (10^3^ cel./μL)	Eosinophils (10^3^ cel./μL)	Basophils (10^3^ cel./μL)
Healthy	Female	SPRET/EiJ	1.89 (0.57)	0.23 (0.13)	0.07 (0.05)
		CD1	1.15 (0.26)	0.06 (0.02)	0.02 (0.00)
	Male	SPRET/EiJ	3.93 (1.14)*	0.15 (0.02)	0.04 (0.01)
		CD1	1.05 (0.09)	0.08 (0.02)	0.02 (0.00)
3rd Week	Female	SPRET/EiJ	5.25 (0.87)*	0.41 (0.2)	0.07 (0.05)
		CD1	1.48 (0.22)	0.13 (0.05)	0.04 (0.02)
	Male	SPRET/EiJ	3.36 (0.34)*	0.14 (0.05)	0.03(0.01)
		CD1	1.38 (0.22)	0.03 (0.01)	0.00 (0.00)
6th Week	Female	SPRET/EiJ	2.80 (0.59)	0.08 (0.03)	0.02 (0.01)
		CD1	1.99 (0.41)	0.05 (0.02)	0.01 (0.00)
	Male	SPRET/EiJ	3.86 (0.29)*	0.06 (0.02)	0.02 (0.00)
		CD1	2.43 (0.24)	0.04 (0.01)	0.01 (0.00)
9th Week	Female	SPRET/EiJ	3.67 (0.75)	0.19 (0.09)	0.02 (0.01)
		CD1	2.05 (0.20)	0.10 (0.03)	0.02 (0.01)
	Male	SPRET/EiJ	3.86 (0.63)	0.06 (0.03)	0.01 (0.00)
		CD1	5.21 (1.00)	0.13 (0.02)	0.01 (0.00)

All results presented as: Mean (SEM). *p < 0.05.

**Table 3.** Summary of blood parameters in *Mus spretus* (SPRET/EiJ strain) and *Mus musculus* (CD1 strain) mice during S*chistosoma mansoni* infection.

Time point	Sex	Mice	White blood cells (10^3^ cel./μL)	Lymphocytes (10^3^ cel./μL)	Monocytes (10^3^ cel./μL)
Healthy	Female	SPRET/EiJ	5.6 (1.4)	2.93 (0.65)	0.52 (0.09)
		CD1	4.95 (0.8)	3.30 (0.60)	0.42 (0.08)
	Male	SPRET/EiJ	6.64 (1.13)	1.95 (0.26)	0.57 (0.07)*
		CD1	5.11 (0.4)	3.61 (0.32)*	0.33 (0.02)
3rd Week	Female	SPRET/EiJ	13.7 (1.9)*	6.94 (0.86)*	0.99 (0.09)*
		CD1	5.62 (0.6)	3.53 (0.26)	0.44 (0.07)
	Male	SPRET/EiJ	5.29 (0.37)	3.22 (0.51)*	0.32 (0.03)
		CD1	4.95 (0.7)	1.43 (0.32)	0.31 (0.03)
6th Week	Female	SPRET/EiJ	6.0 (0.9)	2.55 (0.51)	0.56 (0.03)
		CD1	7.14 (1.14)	4.18 (0.64)	0.896 (0.18)
	Male	SPRET/EiJ	6.8 (0.6)	2.12 (0.27)	0.730 (0.04)
		CD1	8.14 (1.04)	5.07 (0.78)*	0.581 (0.11)
9th Week	Female	SPRET/EiJ	6.51 (1.37)	1.92 (0.51)	0.67 (0.10)
		CD1	5.78 (0.54)	3.16 (0.46)	0.44 (0.03)
	Male	SPRET/EiJ	6.74 (1.74)	2.19 (1.04)	0.60 (0.13)
		CD1	11.24 (1.29)*	5.15 (0.61)*	0.71 (0.10)

All results presented as: Mean (SEM). *p < 0.05.

The present study highlights the importance of *Mus spretus* to unravel mechanisms of resistance/susceptibility against *S. mansoni* infection. The elevated neutrophil counts probably could be a reason for the tolerability of SPRET/EiJ mice against *S. mansoni* infection. Further analysis of the complex host response to *S. mansoni* infection, combined with the recent availability of the complete SPRET/EiJ genome sequence, will contribute to more understanding of the genetic control of host immunity against *S. mansoni* infection and the essential role of neutrophils in early immune defense mechanisms.
